# Death at South Shields Union Infirmary

**Published:** 1914-05-09

**Authors:** 


### Death at South Shields Union Infirmary.

The death of Miss A. A. Zahn, superintendent nurse of the South Shields Union Infirmary, who passed away on Saturday afternoon the 25th ultimo, after a somewhat protracted illness, has been a grief to the whole staff. Miss Zahn had held the post of superintendent nurse for over seven years, having been promoted from that of charge nurse. Miss Zahn endeared herself to all with whom she came in contact. Her devotion to duty and self-sacrifice on behalf of patients and staff still evoke their admiration. She had under her charge a staff of over thirty, and all who were qualified under her tuition will look back upon her work (writes a correspondent) and consideration for them with the deepest satisfaction. The interment took place on the 28th, when a service was held at the Nurses' Home, conducted by Rev. J. Gill, chairman of the Board, and Rev. J. Robson, in whose parish the infirmary is situated. A large number of mourners were present, comprising relatives, friends, officers, and Guardians. The procession subsequently proceeded to Harton Cemetery, accompanied by the medical officer, Dr. Giles, the majority of the nursing staff in uniform, the master and matron, Mr. and Mrs. E. Read, the clerk to the Guardians, Mr. W. J. McCullough, and his assistant clerks, and a number of other officers from the institution. "To live in the hearts of those we leave behind, is not to die," or, as Samuel Butler put it, "where dead men meet, on lips of living men," provides a good three score years and ten of immortality worth earning by personal service in the institutional world.

